# Associations between *TAB2* Gene Polymorphisms and Epithelial Ovarian Cancer in a Chinese Population

**DOI:** 10.1155/2019/8012979

**Published:** 2019-08-14

**Authors:** Xingming Huang, Can Shen, Yan Zhang, Qin Li, Kai Li, Yanyun Wang, Yaping Song, Min Su, Bin Zhou, Wei Wang

**Affiliations:** ^1^Department of Pathology, West China Second University Hospital, Sichuan University, Chengdu, Sichuan 610041, China; ^2^Laboratory of Molecular Translational Medicine, Center for Translational Medicine, Key Laboratory of Birth Defects & Related Diseases of Women & Children (Sichuan University), Ministry of Education, West China Second University Hospital, Sichuan University, Chengdu, Sichuan 610041, China; ^3^Department of Cardiology, West China Hospital of Sichuan University, Chengdu, Sichuan 610041, China; ^4^Department of Immunology, West China School of Preclinical & Forensic Medicine, Sichuan University, Chengdu 610041, Sichuan, China

## Abstract

**Background:**

Epithelial ovarian cancer (EOC) is highly lethal worldwide. Factors involved in the inflammation and hormone-associated signaling pathway play vital roles in EOC carcinogenesis. The transforming growth factor-*β*- (TGF-*β*-) activated kinase 1 (MAP3K7) binding protein 2 (*TAB2*), mediating convergence of inflammatory and estrogen, may be implicated in EOC. The present study is aimed at exploring the association between the *TAB2* gene polymorphisms and EOC.

**Methods:**

Three single nucleotide polymorphisms (SNPs) (rs237028, rs521845, and rs652921) of *TAB2* were genotyped by polymerase chain reaction-restriction fragment length polymorphism (PCR-RFLP) in 221 patients and 252 healthy controls. Associations between SNPs and clinical characteristics were performed either with the *χ*^2^ test or with Fisher's exact test. The Kaplan-Meier method and Cox proportional hazard models were used to detect associations between genotypes and overall survival.

**Results:**

The rs237028 polymorphism was significantly associated with an increased risk of EOC with an allelic genetic model (A *vs.* G; OR = 1.45; 95%CI = 1.07–1.96; *P* = 0.016), dominant genetic model (AA *vs.* AG-GG; OR = 1.66; CI 1.14–2.41; *P* = 0.008), and overdominant genetic model (AA-GG *vs.* AG; OR = 1.60; CI 1.08–2.36; *P* = 0.017). However, no significant association was observed between rs237028 polymorphism and overall survival.

**Conclusions:**

Our study indicated that the rs237028 polymorphism in the *TAB2* gene was associated with EOC susceptibility and the *TAB2* gene might contribute to the initiation of EOC.

## 1. Introduction

Ovarian cancer is the eighth most common malignancy in women. In 2018, there were an estimated 295,414 new cases and 184,799 deaths from ovarian cancer worldwide [1]. Of these, epithelial ovarian cancer (EOC) is the most common cancer, accounting for 90% of all cases [2]. Although ovarian cancer accounts for 3.4% of new cancer cases in females, it also accounts for 4.4% of cancer-related deaths owing to poor five-year survival rates [1]. Late diagnosis at advanced stages is the leading cause of death from ovarian cancer. Therefore, early screening methods are needed to reduce its mortality rates. However, the two most common screening methods, transvaginal ultrasound and measuring serum cancer antigen 125 (CA-125) levels, have failed to show a clinically significant mortality benefit [3, 4]. Hence, analyses of lifestyle-based and genetic risk cofactors are required to identify high-risk populations for appropriate screening. Many lifestyle-based risk factors have already been identified, including childbearing, tubal ligation, oral contraceptive use, and menopausal hormone therapy [5–8]. In addition, several common genetic variants have been found to be risk factors; a combination of polygenic risk scores and epidemiologic risk factors has been identified in genome-wide association studies (GWAS) [9–12]. However, these genetic variants typically rely on the most significant single variants, which account for only slight increases in the prediction of EOC risk, implying that many more potential risk loci need to be identified.

Evidence from various sources has suggested that inflammation contributes to EOC carcinogenesis, and several risk factors associated with inflammation have been found to play a role in the development of EOC [13–15]. Furthermore, SNPs from genes controlling several inflammation-related pathways have been found to have an association with ovarian cancer risk, such as those in the nuclear factor-*κ*B (NF-*κ*B) signaling pathway [16].

Transforming growth factor-*β*- (TGF-*β*-) activated kinase 1 (MAP3K7) binding protein 2 (TAB2), a protein linking TGF-*β*-activated kinase 1 (TAK1) to tumor necrosis factor (TNF) receptor-associated factor 6 (TRAF6) in the TAK1 pathway, mediates the activation of NF-*κ*B [17]. The TAB2 protein, which is encoded by the *TAB2* gene and facilitates the activation of the NF-*κ*B signaling pathway, may play important roles in the development of EOC. Moreover, *TAB2*, which is located on chromosome 6q25.1, has been recognized as a breast cancer risk locus mainly owing to its association with estrogen [18]. Additionally, EOC is a hormone-related tumor that shares many risk factors with breast cancer [19]. However, there are no previous studies investigating the genetic variants of *TAB2* in EOC pathogenesis. Thus, the aim of this study was to assess the association of three SNPs in *TAB2* with EOC susceptibility by genotyping *TAB2* in EOC patients and healthy subjects.

## 2. Materials and Methods

### 2.1. Patients and Subjects

A total of 473 blood samples from 221 EOC patients who underwent surgical resection at the West China Second University Hospital of Sichuan University (China), as well as 252 matched healthy females, were collected between June 2008 and June 2013. The patients were histologically verified to have EOC using the resected specimens. Patients were excluded if they had any other malignancies. The control samples were collected from women aged 29–70 years (mean ± SD: 49.89 ± 11.9 years) who were admitted to the same hospital for routine scheduled physical exams; controls were confirmed to have no serious disease or a family history of major cancers. The study was performed with the approval of the ethics review board of the West China Second University Hospital of Sichuan University (approval no. 2012016), and all the participants gave written informed consent.

### 2.2. DNA Extraction and Genotyping Assays

DNA was isolated from the peripheral blood of patients using a whole blood DNA isolation kit (BioTeke, Beijing, China) and then stored at -20°C for PCR. The following primers were used: rs237028, forward 5′-GCAGACTTGGAAAAGCAAACA-3′ and reverse 5′-CCAGCCTGAGCAACAAGAG-3′; rs521845, forward 5′-TAGGGCGGTTGAGAAGTGAA-3′ and reverse 5′-CCTGGGTGACTGAGCTCTTA-3′; and rs652921, forward 5′-GGCCATTTGGCTCAGAAAT-3′ and reverse 5′-GAGGGAGCTCAGTGGAATTG-3′. PCR was performed at a final reaction volume of 10 *μ*L containing 100 ng DNA, 0.15 *μ*L forward and reverse primers, 1 *μ*L 10x Taq Buffer, and 5 *μ*L 2x Power Taq PCR Master Mix (BioTeke) under the following conditions: 95°C for 4 min, followed by 30 cycles at 94°C for 30 s, 60°C for 30 s, and 72°C for 30 s, with a final extension step at 72°C for 10 min. The genetic polymorphisms of *TAB2* were genotyped using PCR-restriction fragment length polymorphism (PCR-RFLP). The PCR products of rs237028, rs521845, and rs652921 were digested using the restriction enzymes Hpy188I (2 h at 37°C), Psp1406I (16 h at 37°C), and BseDI (45 min at 37°C), respectively. Next, 5 *μ*L digested PCR products were isolated on a 6% polyacrylamide gel and stained with silver nitrate. The genotypes of rs237028 were designated as follows: A/A, a single band consisting of 138 bp; A/G, three bands consisting of 138 bp, 106 bp, and 32 bp; and G/G, two bands consisting of 106 bp and 32 bp. Similarly, for rs521845, the 120 bp and 100 bp fragments were, respectively, designated as allele T and G; and for rs652921, the 120 bp and 100 bp fragments were, respectively, designated as allele C and T.

### 2.3. Clinical Information and Follow-Up

Patients were excluded from survival analysis if follow-up information was not available. Ultimately, 140 patients were eligible for survival analysis. The clinical information of the patients was obtained from medical records. The overall survival was defined as the time from diagnosis to the last follow-up date or the date of death. The clinical stage and grade of the tumors were determined based on the criteria of the International Federation of Obstetrics and Gynecology (FIGO) and WHO [20, 21].

### 2.4. Statistical Analyses

The Hardy-Weinberg equilibrium (HWE) and the association between *TAB2* gene polymorphisms and EOC were calculated by SNPstats online software (http://www.snpstats.net/start.htm), which assessed the frequency distributions between patients and healthy controls in four genetic models: codominant, dominant, recessive, and overdominant [22]. The association between rs237028 and clinical characteristics was analyzed using either the *χ*^2^ test or Fisher's exact test. Survival curves were analyzed by the Kaplan-Meier and log-rank test, and multivariable overall survival analysis was performed using Cox proportional hazard models. All data were analyzed using SPSS 22.0 (SPSS Inc., Chicago, IL, USA). Differences were considered statistically significant at a value of *P* < 0.05.

## 3. Results

### 3.1. Subject Characteristics

A total of 221 EOC patients and 252 healthy female controls were investigated in this study. The clinical characteristics of the patients are shown in Table 1.

### 3.2. *TAB2* SNPs in EOC

In this study, the genotypes of three SNPs (rs237028, rs521845, and rs652921) in the patients and controls followed the HWE. The distributions of the allelic and genotypic frequencies are shown in Table 2. For rs237028, the frequencies of the AA, AG, and GG genotypes, respectively, were 66.4%, 28.1%, and 5.5% in patients and 54.4%, 38.5%, and 7.1% in controls. The distribution of the SNPs between EOC patients and the control group was significantly different based on the codominant (AG vs. GG; odds ratio (OR) = 1.58; 95% confidence interval (95% CI): 0.73–3.39; *P* = 0.030), dominant (AA vs. AG-GG; OR = 1.66; 95% CI: 1.14–2.41; *P* = 0.008), and overdominant genetic models (AA-GG vs. AG; OR = 1.60; 95% CI: 1.08–2.36; *P* = 0.017), as well as in terms of allele frequencies (A vs. G; OR = 1.45; 95% CI: 1.07–1.96; *P* = 0.016). No significant associations were observed for rs521845 or rs652921.

### 3.3. Association between rs237028 and Clinical Characteristics

Stratification analyses were performed to evaluate the associations between each genetic polymorphism and clinical characteristics. As illustrated in Table 3, the A allele of rs237028 was associated with increased age (>50 years; OR = 0.544; 95% CI: 0.296–1.001; *P* = 0.049) and higher tumor grade (G2 and G3; OR = 2.664; 95% CI: 1.143–6.211; *P* = 0.020). There were no significant associations between rs237028 and other clinical characteristics, such as FIGO stage, histology type, tumor position, lymph node status, peritoneum invasion, or vascular invasion.

### 3.4. Survival Analysis

**Figure 1:**
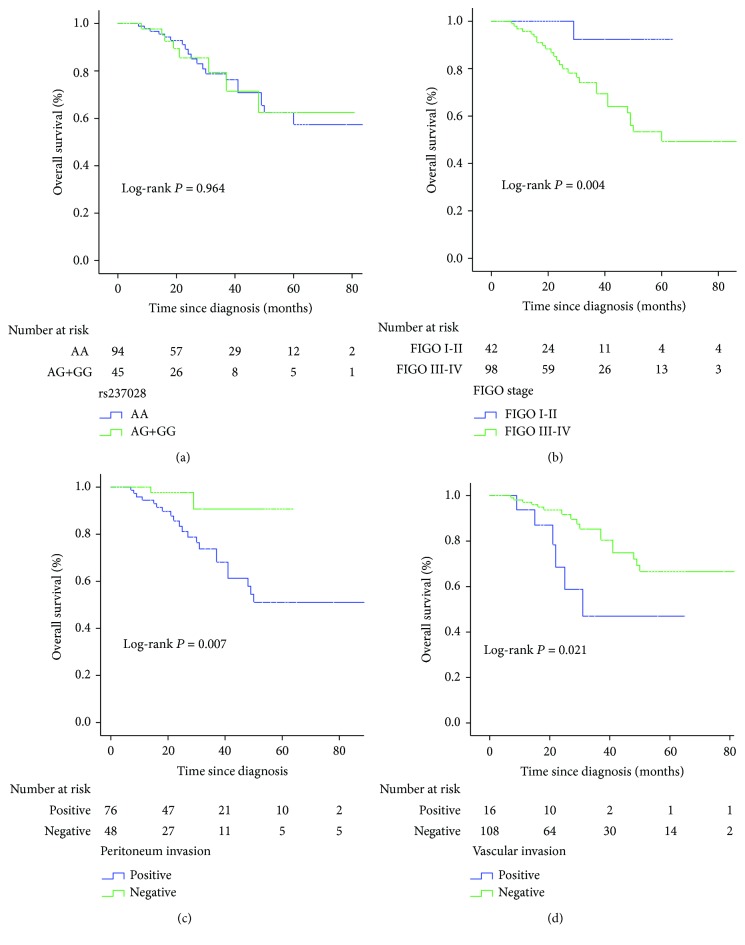
Kaplan-Meier analysis for overall survival in EOC patients. (a) The SNP rs237028. (b) FIGO stage. (c) Peritoneum invasion. (d) Vascular invasion. FIGO = Federation International of Gynecology and Obstetrics.

To evaluate the association of rs237028 and the prognosis of EOC patients, we conducted survival analysis for 140 patients. The Kaplan-Meier curves showed no significant associations between rs237028 and overall survival (AA vs. AG-GG; *P* = 0.964). The prognostic factors in our study, including advanced stage (FIGO III–IV; *P* = 0.004), peritoneum invasion (*P* = 0.007), and vascular invasion (*P* = 0.021), were significantly associated with a poor outcome; however, this association was not observed for age, grade histology, tumor position, or lymph node state. The Kaplan-Meier curves for overall survival in EOC patients are shown in Figure 1. According to multivariate Cox regression analysis, the prognostic value was only shown at advanced stages (FIGO III–IV; 95% CI: 1.015–64.914; *P* = 0.048).

## 4. Discussion

In this study, we investigated the role of genetic variants of *TAB2* in EOC. To our knowledge, the present study is the first to confirm that the rs237028 variant of *TAB2* is associated with susceptibility to EOC; however, the other two genetic variants, rs521845 and rs652921, showed no associations with EOC. Additionally, patients with the rs237028 A allele tended to be older (>50 years) and had higher-grade tumors (G2 and G3) compared to patients with the G allele.

The rs237028 SNP is located in an intron of the *TAB2* gene, which encodes the protein TAB2. TAB2 was originally recognized as an adaptor protein for TAK1 signaling; it acts by tethering TAK1 to the polyubiquitinated protein TRAF6, which in turn facilitates the activation of inhibitor of *κ*B kinase (IKK) and NF-*κ*B. Constitutive NF-*κ*B signaling plays a vital role in tumorigenesis and metastasis in various types of cancers, including EOC [23]. Yung et al. recently reported that the TAK1/NF-*κ*B expression and signaling were increased in ovarian metastatic cancer cells and that treatment targeting TAK1/NF-*κ*B signaling significantly decreased the oncogenic and metastatic potential of the cancer cells [24]. Additionally, Chen et al. found that the transforming growth factor-*β*- (TGF-*β*-) activated kinase 1 (MAP3K7) binding protein 2 (TAB3), a homolog of TAB2 that is highly expressed in EOC cells and tissues, appears to play a role in accelerating EOC development [25]. Further evidence has suggested that SNPs related to NF-*κ*B activation are associated with increased EOC risk [16]. The findings of the present study were consistent with this conclusion. In our study, we concluded that patients with the AA or AG genotypes of the rs237028 SNP had a higher risk of developing EOC than patients with the GG genotype. Notably, SNPs associated with disease risk are typically found in the non-protein-coding genome, and they function to influence gene expression by altering the non-protein-coding genome [26]. Thus, we proposed that the rs237028 risk allele, located in an intron of the *TAB2* gene, may facilitate EOC development by dysregulating the function of the intron region to influence the TAK1/NF-*κ*B signaling pathway.

In addition to its role as an important component in the inflammatory pathway, *TAB2*, which is located on chromosome 6q25.1 near the estrogen receptor 1 (*ESR1*) gene, is considered to be a risk factor for hormone-related cancers owing to its role in the regulation of the *ESR1* expression. Li et al. found that *ESR1* and *TAB2* expression levels were decreased in hepatocellular carcinomas compared to those in adjacent normal tissues and that the *ESR1* expression was significantly associated with the expression of *TAB2* [27]. In a previous study, both *TAB2* and *ESR1* gene polymorphisms were found to be associated with breast cancer [18]. In addition, some researchers have suggested that TAB2, by interacting with the domain of estrogen receptor *α* (ER*α*), suppresses the transcriptional activity of nuclear receptor corepressor (NCoR), thus rescuing the repression of estrogen receptor (ER) signaling [28]. Notably, EOC, hepatocellular carcinoma, and breast cancer are collectively known as hormone-related cancers; several risk factors that are recognized to have exogenous or endogenous influences on estrogen exposure in breast cancer have also been shown to affect EOC [29]. In fact, GWAS have identified several risk loci demonstrating a shared association between breast and ovarian cancer risk [19]. Previous studies have also shown that *ESR1* is involved in the development and progression of EOC [30], and genetic susceptibility studies have suggested that polymorphisms in the *ER-α* gene confer increased risk for EOC [31]. Thus, in line with the present study, it is possible that the association between rs237028 and EOC susceptibility be mediated through the ER signaling pathway. However, defining the underlying mechanism of the rs237028 SNP in the development of EOC required further detailed functional investigations.

The present study also indicated that older patients (>50 years) were more likely to have the rs237028 A allele. Generally, older patients were also found to be undergoing menopause, and their estrogen levels were therefore considerably lower than those in premenopausal women. Accordingly, this result may indicate an underlying connection between TAB2 and the ER signaling pathway to stimulate the development of EOC. Moreover, the activation of the TAK1/NF-*κ*B pathway affects the production of steroid hormones and may contribute to the pathogenesis of EOC. Collectively, our evidence suggests that TAB2 is a central factor with a role in several pathways involved in inflammation and estrogen production; it is therefore likely associated with EOC and may play a role in EOC initiation and progression. Although this study identified associations between the rs237028 SNP and susceptibility to EOC, no significant associations were observed between any polymorphisms of *TAB2* and overall survival, as shown by univariate and multivariate overall survival analysis. The rs237028 SNP seems to act as a risk factor for the development of EOC but is not a predictive factor for cancer prognosis. Moreover, our analysis of the underlying factors affecting overall survival in EOC, including age, FIGO stage, grade, histology, tumor position, lymph node status, peritoneum invasion, and vascular invasion showed that only FIGO stage, peritoneum invasion, and vascular invasion had a significant association with EOC.

This study has several limitations. Firstly, the clinical information of some patients was missing, although this part of missing was randomized, and these patients were not included in survival analysis. But the limited sample size may have affected the veracity and objectivity of our results. Therefore, these findings need to be further corroborated in larger cohorts. Secondly, the functions of the three SNPs investigated in this research are unclear, and the underlying mechanisms of the rs237028 SNP in the development of EOC remain poorly understood. Further investigations are warranted.

## 5. Conclusions

Taken together, our study identified a potential EOC susceptibility locus, and through stratified analysis, the A allele of rs237028 was found to be significantly associated with increased age and higher tumor grade in EOC. The identification of multiple genetic polymorphisms with effects on susceptibility in many diseases, as well as the important roles of *TAB2* in the pathogenesis of EOC based on previous research, led us to conclude that rs237028 in *TAB2* potentially plays a major role in the development of EOC. Thus, with the exception of several lifestyle-based risk factors that have been recognized for several years, *TAB2* may be used as a potential marker for screening high-risk populations for EOC.

## Figures and Tables

**Table 1:** Descriptive characteristics of the epithelial ovarian cancer patients.

Characteristics	Value
Sample size	*N* = 140
Mean age ± SD, range (years)	48.89 ± 10.43, 15-71
FIGO stage, number (%)	
I	31 (22.1)
II	11 (7.9)
III	90 (64.3)
IV	8 (5.7)
Tumor grade, number (%)	
G1	12 (8.6)
G2	19 (13.6)
G3	96 (68.6)
Unknown^∗^	13 (9.3)
Histology, number (%)	
Serous	76 (54.3)
Clear cell	16 (11.4)
Endometrioid	8 (5.7)
Mucinous	9 (6.4)
Mixed and other	31 (22.1)

SD = standard deviation, FIGO = International Federation of Gynecology and Obstetrics. ^∗^Clear cell carcinoma.

**Table 2:** Genotype and allele distribution of TAB2 gene polymorphisms in patients with epithelial ovarian cancer and healthy controls.

Genotype or allele		Case *N* = 221 (%)	Control *N* = 252 (%)	OR (95% CI)	*P* value
rs237028 Genetic model	Genotype				
Codominant	A/A	144 (66.4)	137 (54.4)	1.00	0.03^∗^
	A/G	61 (28.1)	97 (38.5)	1.67 (1.12-2.49)	
	G/G	12 (5.5)	18 (7.1)	1.58 (0.73-3.39)	
Dominant	A/A	144 (66.4)	137 (54.4)	1.00	0.008^∗^
	A/G-G/G	73 (33.6)	115 (45.6)	1.66 (1.14-2.41)	
Recessive	A/A-A/G	205 (94.5)	234 (92.9)	1.00	0.47
	G/G	12 (5.5)	18 (7.1)	1.31 (0.62-2.79)	
Overdominant	A/A-G/G	156 (71.9)	155 (61.5)	1.00	0.017^∗^
	A/G	61 (28.1)	97 (38.5)	1.60 (1.08-2.36)	
Allele	A	349 (80)	371 (74)		0.016^∗^
	G	85 (20)	133 (26)	1.45 (1.07-1.96)	
rs521845 Genetic model					
Codominant	T/T	85 (40.5)	105 (41.7)	1.00	0.97
	T/G	100 (47.6)	118 (46.8)	0.96 (0.65-1.41)	
	G/G	25 (11.9)	29 (11.5)	0.94 (0.51-1.72)	
Dominant	T/T	85 (40.5)	105 (41.7)	1.00	0.80
	T/G-G/G	125 (59.5)	147 (58.3)	0.95 (0.66-1.38)	
Recessive	T/T-T/G	185 (88.1)	223 (88.5)	1.00	0.89
	G/G	25 (11.9)	29 (11.5)	0.96 (0.54-1.70)	
Overdominant	T/T-G/G	110 (52.4)	134 (53.2)	1.00	0.86
	T/G	100 (47.6)	118 (46.8)	0.97 (0.67-1.40)	
Allele	T	270 (64)	328 (65)	0.96 (0.73-1.27)	0.8
	G	150 (36)	176 (35)		
rs652921 Genetic model					
Codominant	T/T	66 (32.5)	85 (33.7)	1.00	0.53
	T/C	87 (42.9)	116 (46)	1.04 (0.68-1.58)	
	C/C	50 (24.6)	51 (20.2)	0.79 (0.48-1.31)	
Dominant	T/T	66 (32.5)	85 (33.7)	1.00	0.78
	T/C-C/C	137 (67.5)	167 (66.3)	0.95 (0.64-1.40)	
Recessive	T/T-T/C	153 (75.4)	201 (79.8)	1.00	0.26
	C/C	50 (24.6)	51 (20.2)	0.78 (0.50-1.21)	
Overdominant	T/T-C/C	116 (57.1)	136 (54)	1.00	0.5
	T/T	87 (42.9)	116 (46)	1.14 (0.78-1.65)	
Allele	T	219 (54)	286 (57)	0.90 (0.70-1.16)	0.42
	C	187 (46)	218 (43)		

CI = confidence interval, OR = odds ratio. ^∗^*P* < 0.05.

**Table 3:** Association between the genotype distribution of rs237028 and characteristics of the EOC patients.

Clinical features	Genotype			Genetic model								Allele			
				Codominant		Dominant		Recessive		Overdominant					
				(A/A vs. A/G vs G/G)		(A/A vs. A/G-G/G)		(A/A-A/G vs. G/G)		(A/A-G/G vs. A/G)					
	A/A	A/G	G/G	OR (95% CI)	*P* value	OR (95% CI)	*P* value	OR (95% CI)	*P* value	OR (95% CI)	*P* value	A	G	OR (95% CI)	*P* value
Age															
<50	41	20	6	A/G: 0.658 (0.306-1.412)	0.281	0.565 (0.276-1.160)	0.118	0.290 (0.057-1.493)	0.115	0.726 (0.341-1.545)	O.406	102	32	0.544 (0.296-1.001)	0.049^∗^
≥50	53	17	2	G/G: 0.258 (0.049-1.345)	0.090							123	21		
FIGO stage															
I-II	31	8	2	A/G: 1.784 (0.730-4.357)	0.201	1.722 (0.756-3.926)	0.193	1.272 (0.246-6.579)	0.564	1.734 (0.715-4.204)	0.220	70	12	1.543 (0.764-3.115)	0.224
III-IV	63	29	6	G/G: 1.476 (0.282-7.741)	0.489							155	41		
Grade															
G1	6	5	1	A/G: 2.194 (0.623-7.729)	0.182	2.194 (0.662-7.273)	0.161	1.652 (0.182-14.994)	0.510	2.024 (0.597-6.860)	0.205	18	10	2.664 (1.143-6.211)	0.020^∗^
G2-G3	79	30	6	G/G: 2.194 (0.226-21.324)	0.437							187	39		
Histology															
Serous	49	20	5	A/G: 1.056 (0.492-2.286)	0.888	1.122 (0.549-2.295)	0.752	1.473 (0.338-6.423)	0.443	1.024 (0.481-2.180)	0.951	118	30	1.183 (0.647-2.161)	0.585
Others	44	17	3	G/G: 1.497 (0.338-6.628)	0.438							107	23		
Tumor position															
Unilateral	27	7	3	A/G: 1.815 (0.693-4.753)	0.221	1.429 (0.603-3.387)	0.276	0.453 (0.087-2.362)	0.293	1.905 (0.735-4.939)	0.181	61	13	1.117 (0.544-2.293)	0.762
Bilateral	51	24	3	G/G: 0.529 (0.100-2.804)	0.365							126	30		
Lymph node status															
Negative	36	16	5	A/G: 0.622 (0.228-1.696)	0.352	0.681 (0.266-1.743)	0.421	1.154 (0.209-6.377)	0.619	0.624 (0.235-1.662)	0.344	88	26	0.802 (0.378-1.703)	0.565
Positive	14	10	2	G/G: 0.972 (0.169-5.607)	0.643							38	14		
Peritoneum invasion															
Negative	32	13	3	A/G: 0.905 (0.399-2.050)	0.811	0.907 (0.423-1.944)	0.803	0.947 (0.216-4.156)	0.627	0.912 (0.407-2.043)	0.495	77	19	0.925 (0.490-1.747)	0.811
Positive	49	22	5	G/G: 0.919 (0.205-4.114)	0.613							120	32		
Vascular invasion															
Negative	72	28	8	A/G: 0.500 (0.170-1.472)	0.153	0.643 (0.221-1.866)	0.441	1.080 (1.024-1.139)	0.320	0.450 (0.153-1.322)	0.121	172	44	0.914 (0.371-2.250)	0.844
Positive	9	7	0	G/G: 1.111 (1.033-1.195)	0.410							25	7		

CI = confidence interval, FIGO = International Federation of Gynecology and Obstetrics, OR = odds ratio. ^∗^*P* < 0.05.

## Data Availability

The data used to support the findings of this study are included within the article.
